# The VirF_21_:VirF_30_ protein ratio is affected by temperature and impacts *Shigella flexneri* host cell invasion

**DOI:** 10.1093/femsle/fnac043

**Published:** 2022-05-06

**Authors:** Eva Skovajsová, Bianca Colonna, Gianni Prosseda, Mikael E Sellin, Maria Letizia Di Martino

**Affiliations:** Science for Life Laboratory, Department of Medical Biochemistry and Microbiology, Uppsala University, 75123, Sweden; Department of Biology and Biotechnology “C. Darwin”, Istituto Pasteur Italia, Sapienza Università di Roma, Rome, 00185, Italy; Department of Biology and Biotechnology “C. Darwin”, Istituto Pasteur Italia, Sapienza Università di Roma, Rome, 00185, Italy; Science for Life Laboratory, Department of Medical Biochemistry and Microbiology, Uppsala University, 75123, Sweden; Science for Life Laboratory, Department of Medical Biochemistry and Microbiology, Uppsala University, 75123, Sweden

**Keywords:** *Shigella*, virulence genes, regulation, infection, cell invasion, Shigellosis

## Abstract

*Shigella spp*, the etiological agents of bacillary dysentery in humans, have evolved an intricate regulatory strategy to ensure fine-tuned expression of virulence genes in response to environmental stimuli. A key component in this regulation is VirF, an AraC-like transcription factor, which at the host temperature (37°C) triggers, directly or indirectly, the expression of > 30 virulence genes important for invasion of the intestinal epithelium. Previous work identified two different forms of VirF with distinct functions: VirF_30_ activates virulence gene expression, while VirF_21_ appears to negatively regulate *virF* itself. Moreover, VirF_21_ originates from either differential translation of the *virF* mRNA or from a shorter leaderless mRNA (llmRNA). Here we report that both expression of the *virF_21_* llmRNA and the VirF_21_:VirF_30_ protein ratio are higher at 30°C than at 37°C, suggesting a possible involvement of VirF_21_ in minimizing virulence gene expression outside the host (30°C). Ectopic elevation of VirF_21_ levels at 37°C indeed suppresses *Shigella*´s ability to infect epithelial cells. Finally, we find that the VirF_21_ C-terminal portion, predicted to contain a Helix-Turn-Helix motif (HTH2), is required for the functionality of this negative virulence regulator.

## Teaser

The ratio of the two virulence-regulatory proteins VirF_21_:VirF_30_ increases at low temperature and can dictate the invasive properties of *Shigella flexneri*.

## Introduction

Enterobacterial pathogens coordinate the expression of virulence factors through complex regulatory networks in order to colonize and disseminate in the host gut epithelium. *Shigella flexneri* bacteria, facultative intracellular microbes causing bacillary dysentery in humans, have become paradigmatic for the study of virulence gene regulation. The expression and combined action of numerous virulence factors, mainly encoded on a large virulence plasmid (pINV), ultimately result in the invasion of colonic epithelial cells in the lower gut (Mattock and Blocker 2017). Subsequently, the bacteria multiply intracellularly and spread to adjacent cells, resulting in cell death and inflammatory destruction of the gut mucosa (Schroeder and Hilbi 2008, Arena *et al*. 2015). A crucial regulator of the *Shigella* infection process is VirF, an AraC-like transcription factor, responsible for the invasive phenotype (Di Martino *et al*. 2016a). The synthesis of VirF occurs when *Shigella* senses the transition from the external environment to the human host (Falconi *et al*. 1998, Prosseda *et al*. 2004). VirF then triggers a regulatory cascade involving the expression of *virB* and *icsA* genes (Tobe *et al*. 1993, Tran *et al*. 2011). VirB activates a second wave of virulence genes involved in the assembly of a type 3 secretion system (T3SS), its effectors (the ipa-spa operons), and a second AraC-like transcriptional activator, *mxiE* (Le Gall *et al*. 2005, Parsot 2005, Schroeder and Hilbi 2008). IcsA, on the other hand, promotes *Shigella* dissemination across adjacent cells through host actin polymerization (Bernardini *et al*. 1989, Lett *et al*. 1989). Finally, the master regulator VirF also activates some chromosomally located genes (e.g; the spermidine excretion complex MdtJI; the chaperones IbpA, HtpG, DnaK and the protease Lon) whose expression may optimize *Shigella*´s intracellular life style (Barbagallo *et al*. 2011, Leuzzi *et al*. 2015).

The activation of *virF* is a key event for the successful invasion and dissemination of *Shigella* within the host epithelium, and is therefore stringently regulated. A multitude of environmental signals (e.g. temperature, pH, osmolarity) affect *virF* expression through several regulatory mechanisms. Some of these mechanisms have been described at the molecular level, as the temperature-dependent expression of the *virF* gene (Falconi *et al*. 1998, Prosseda *et al*. 2004). At temperatures below 32°C (non-permissive), the nucleoid-associated protein H-NS tightly binds two sites within the *virF* promoter. This prevents access of the RNA polymerase and therefore leads to *virF* transcriptional silencing. At the permissive host temperature (37°C), relaxation of an intrinsically-curved DNA region, located between the two H-NS binding sites, hampers H-NS binding and favors access of the nucleoid associated protein FIS to one of its binding sites (Falconi *et al*. 2001, Prosseda *et al*. 2004). This results in activation of *virF* transcription. VirF subsequently acts as an anti-silencer, counteracting H-NS-mediated repression on for example *virB* and *icsA* promoters. Besides direct binding to the *icsA* promoter, VirF also stimulates *icsA* expression by lowering the intracellular concentration of the antisense rna RnaG, known to cause premature *icsA* transcriptional termination (Tran *et al*. 2011).

In addition to temperature-dependent regulation of *virF* expression, several other regulatory mechanisms have been described, involving e.g. IHF (Porter and Dorman 1997), CpxA/R (Nakayama and Watanabe 1998), and EnvZ/OmpR (Bernardini *et al*. 1990), as well as specific post-transcriptional tRNA modifications (Durand *et al*. 1997, 2000, Hurt *et al*. 2007). More recently, a chromosomal LysR-like transcriptional regulator, YhjC, has been shown to activate *virF* expression, suggesting further cross-talk between chromosomal and pINV located genes (Li *et al*. 2021). Altogether, these factors contribute to reach a fine-tuned VirF threshold concentration, sufficient to activate *virB* and *icsA* expression and thereby trigger the *Shigella* host cell invasive program (Adler *et al*. 1989, Dagberg and Uhlin 1992, Prosseda *et al*. 1998).

Previously, the existence of an additional layer in the regulation of *virF* expression was discovered. The *virF* mRNA itself, through differential translation, in fact gives rise to two forms of VirF protein: VirF_30_ (30 kDa) and VirF_21_ (21 kDa). VirF_30_ acts as primary activator of the virulence gene cascade, whereas VirF_21_ appears to negatively autoregulate *virF* expression through direct promoter binding (Di Martino *et al*. 2016b). In addition, VirF_21_ can originate also from a shorter, leaderless mRNA (llmRNA), transcribed from a gene-internal promoter (Di Martino *et al*. 2016b). While the molecular interactions of this regulatory loop were defined in the previous study, it had remained unknown which conditions affect VirF_21_ expression and how this can impact *Shigella*´s host cell invasive phenotype.

Here we characterized the conditions governing *virF_21_* llmRNA expression and the overall VirF_21_:VirF_30_ protein ratio. We found that at 30°C, a common condition *Shigella* encounters during its extracellular (non-invasive) lifestyle, transcription of the *virF_21_* llmRNA from the internal promoter is favored and the VirF_21_:VirF_30_ protein ratio is elevated, as compared to the permissive host temperature of 37°C. Moreover, ectopically elevating VirF_21_ levels at 37°C resulted in a marked and reversible block of the *Shigella* host cell invasive phenotype. The C-terminal part of VirF_21_ was found to be required for this suppression. We discuss the possible connections between environmental sensing, the fitness costs of virulence gene expression, and VirF_21_-dependent suppression of the host cell invasive program.

## Material and methods

### Bacterial strains and general procedures

Strains and plasmids used in this study are listed in Table S1. M90T is a *S. flexneri* serotype 5 strain (Sansonetti et al 1982). Strain M90T *ΔvirF* carries a deletion of the *virF* gene (Leuzzi *et al*. 2015). Strain M90T *virF-3xFT* carries the 3xFLAG tag sequence at the C-terminus of the pINV-encoded *virF* gene (Leuzzi *et al*. 2015). Strain M90T *ΔmxiD* carries a deletion of the *mxiD* gene and has been constructed using the one-step gene inactivation method (Datsenko and Wanner 2000), transforming M90T pKD46 with the PCR product obtained using plasmid pKD4 as template and the oligo pairs mxiD_delF/mxiD_delR (Table S2). The plasmids pControl (previously named pGIP7), pVirF_21_ (previously named pAC-21), pRS-F(+205) and pRS-F(+305) were described previously (Di Martino *et al*. 2016b, Falconi *et al*. 2001).

The plasmids pVirF_21__I97N, pVirF_21__V108A, pVirF_21__V145T and pVirF_21__Y141stop were obtained by Gibson Assembly (Gibson *et al*. 2009), ligating *in vitro* synthesized PCR products (purchased from Genewiz) into the BamHI restriction site in pControl using the Gibson Assembly Cloning kit from NEB (#E5510S). Amino acid substitutions were adjusted according to the *Shigella* codon usage. The resulting plasmids were transformed into the wild-type M90T strain. Sequences of the PCR products used are listed in Table S2. Bacteria were routinely grown in LB medium at 37°C, unless otherwise specified. When required, strains were grown in M9 complete medium (M9 minimal medium supplemented with 10 mg/ml thiamine, 0.2% glucose, 0.5% casamino acids and 10 mg/ml nicotinic acid). When necessary, antibiotics were supplemented at the following concentrations: ampicillin, 50 µg/ml; chloramphenicol, 15 µg/ml; kanamycin, 50 µg/ml; streptomycin, 50 µg/ml. Plasmid DNA extraction, DNA transformation, electrophoresis, purification of DNA fragments and sequencing were performed as described previously (Green and Sambrook 2012). PCR reactions were performed using DreamTaq DNA polymerase (Thermo Fisher Scientific, #EP0702) or Phusion DNA polymerase (Thermo Fisher Scientific, #F-530L). All oligonucleotide primers used in this study are listed in Table S2.

### ß-galactosidase assays

β-galactosidase assays were performed as previously described (Miller 1992) on sodium dodecyl sulphate-chloroform-permeabilized cells grown in LB or M9 medium (to OD600 0.5–0.6). β-galactosidase activity of the pRS-F (+205) and pRS-F (+305) transcriptional fusions was assessed under different conditions. For temperature shift, ON cultures grown at 30°C in LB were subcultured 1:100 in LB at 30°C or 37°C. To mimic intestine-like conditions, ON cultures grown at 30°C were subcultured 1:100 at 37°C in M9 medium supplemented with the following compounds: Sodium deoxycholate (0, 2.5, 5 mg/ml), Bile salts (0, 6, 9 mg/ml), NaCl (0, 0.1, 0.2M), Hydrogen peroxide (0, 10, 50, 100 mM). To screen different pH conditions, bacteria were subcultured 1:100 in M9 medium at pH 5, 6 and 7.

### Immunodetection of VirF proteins

VirF protein levels were detected by western blot through enhanced chemiluminescence. In brief, equal amount of proteins was extracted from strains grown at OD600 ∼0.6, separated on Any kD™ Mini-PROTEAN® TGX Stain-Free™ Protein Gels (Biorad, #4568126) and transferred onto Trans-Blot Turbo Mini 0.2 µm PVDF Transfer Packs (Biorad, **#**1704156). The stain-free method was used to obtain the loading control (Colella *et al*. 2012). The method is based on the fluorescent detection of tryptophan residues in the protein sequence, as a result of the presence of a trihalo compound in the gel. After protein separation by electrophoresis, each gel was imaged upon exposure to UV-light for 5 min and the same region was selected as loading control for all western blots. Immunodetection was performed as described in Di Martino *et al*. 2016b using polyclonal halon anti-VirF, anti-FLAG (Sigma, #F1804) and anti-GroEL (Sigma, #A8705) antibodies. Quantification by Western blots were obtained by serial dilution of protein extracts, with the relative amounts calculated from a standard curve. For the protein extracts derived from cultures grown at 30°C, concentrated samples were used to calculate the standard curves.

### Congo red binding assay

Congo Red (CR) plates were prepared adding 0.108 mg/ml of Congo Red dye (Sigma, #C6277) to Trypticase Soy Agar (Sigma, #22091) and supplemented with 0, 0.1, 0.25, or 1 mM IPTG (Sigma, #I6758). The indicated *Shigella flexneri* M90T strains were grown ON at 30°C with appropriate antibiotics, diluted 1:40 and subcultured at 37°C for ∼2 h (OD600 ∼0.7). Subcultures were serially diluted and plated on CR plates containing increasing concentrations of IPTG. The following day Congo Red positive (CR+) and negative (CR−) colonies were enumerated. Subsequently, colonies were scraped from each plate, serially diluted and plated on new CR plates without the IPTG selection.

### Epithelial cell culture and infections

Caco-2 cells were grown in DMEM GlutaMAX (Gibco, #31966–021) supplemented with 10% heat-inactivated fetal bovine serum (FBS; Gibco, #10270106) and 0.1 mM Non Essential Amino Acids (Gibco, #11140035) at 37°C with 10% CO_2_. Cultures were passaged two to three times/week in the presence of 100IU/ml penicillin and 100 µg/ml streptomycin, but antibiotics were omitted during infection experiments. Caco-2 cells were seeded in 12-well plates 24–48 h before infection. The indicated *S. flexneri* strains were grown ON at 30°C in LB with appropriate antibiotics, diluted 1:50 in the presence of 0.25 mM IPTG to allow the induction of VirF_21_. The subcultures were further incubated for 2h at 37°C or for 1 h at 30°C before shifting for 1.5 h to 37°C (OD600 ∼0.7). Upon infection, bacteria were centrifuged on top of the cultured epithelial cells for 15 min at 700 g, followed by 45min incubation at 37°C and 10% CO_2_. The culture medium was replaced with fresh medium containing 200 µg/ml Gentamicin (Sigma, #G1914) and the cells were further incubated for 2 h. At 3h post-infection (p.i.) cells were washed and lysed adding 0.1% Sodium deoxycholate, the lysates were then diluted and plated on LB agar plates with appropriate antibiotics, followed by enumeration of the number of colony-forming units (CFUs).

### qRT-PCR

Total RNA purification and cDNA synthesis were performed as previously described (Di Martino *et al*. 2016b). qRT-PCR was performed using Maxima SYBR green/ROX qPCR master mix (2X) (Thermo Fisher Scientific, #K0222) on a CFX384 Touch Real-Time PCR Detection System (Biorad). The levels of *virF*, *virF_30_*, *virB*, *mxiE* and *icsA* transcripts were analysed using the 2^−ΔΔ^*^CT^* (cycle threshold [*CT*]) method and results are reported as the fold increase relative to the reference (Livak *et al*. 2001). The housekeeping gene *nusA* was used for normalization. The following oligonucleotide primers were used (see Table S2): *nusAQF/nusAQR*, virFQF/virFQR, virF30QF/virF30QR, *virBQF/virBQR*, *mxiEQF/mxiEQR* and *icsAQL/icsAQR*.

## Results and discussion

### Expression levels of the *virF_21_* llmRNA and the VirF_21_:VirF_30_ protein ratio are both elevated at non-permissive temperature (30°C)

Previous work identified a *virF_21_* translationally capable llmRNA, whose transcription is dependent on the presence of an internal promoter located two nucleotides upstream the VirF_21_ translational start site (Di Martino *et al*. 2016b). To determine how and when expression of the *virF_21_* llmRNA occurs, *E. coli* strains harbouring a *virF_21_*(llmRNA)-*lacZ* transcriptional fusion construct (pRS-F (+205)), or a control fusion (pRS-F (+305)) (Di Martino *et al*. 2016b) were grown under different conditions, mimicking either the environment *Shigella* encounters during host cell invasion in the gut (i.e. exposure to sodium deoxycholate, bile salts, high osmolarity, oxidative stress, low pH) or the environment outside the host (non-permissive temperature: 30°C) (Marteyn *et al*. 2012). No difference in the pRS-F (+205) ß-galactosidase activity was observed during osmotic, oxidative and pH-stress, as compared to the untreated control (Fig S1A-B-C). Increasing concentrations of either sodium deoxycholate alone or a bile salt mixture, hence mimicking the biliary secretions encountered in the human intestinal tract (Faherty *et al*. 2012, de Buy Wenniger *et al*. 2013, Di Ciaula *et al*. 2017, Nickerson *et al*. 2017, Chanin *et al*. 2019), resulted in ∼6 and ∼3-fold increase in ß-galactosidase activity, respectively (Fig S1D-E). This suggests an increase in *virF_21_* llmRNA transcription under these two conditions. However, the relative abundance of VirF_21_ protein, measured in a *Shigella flexneri* M90T strain harbouring a 3xFlag-tagged version of the VirF proteins, did not increase accordingly in response to these stimuli (Fig S1F-G-H). This may imply that under the conditions tested here, the majority of VirF_21_ protein originates from alternative translation of the *virF* full length mRNA. The existence of some other unknown post-transcriptional regulatory mechanisms, hampering VirF_21_ translation, can also not be ruled out. In either case, typical conditions encountered by *Shigella* within the host may influence *virF_21_* llmRNA transcription, but do not seem to significantly alter VirF_21_ protein levels.

**Figure 1.**
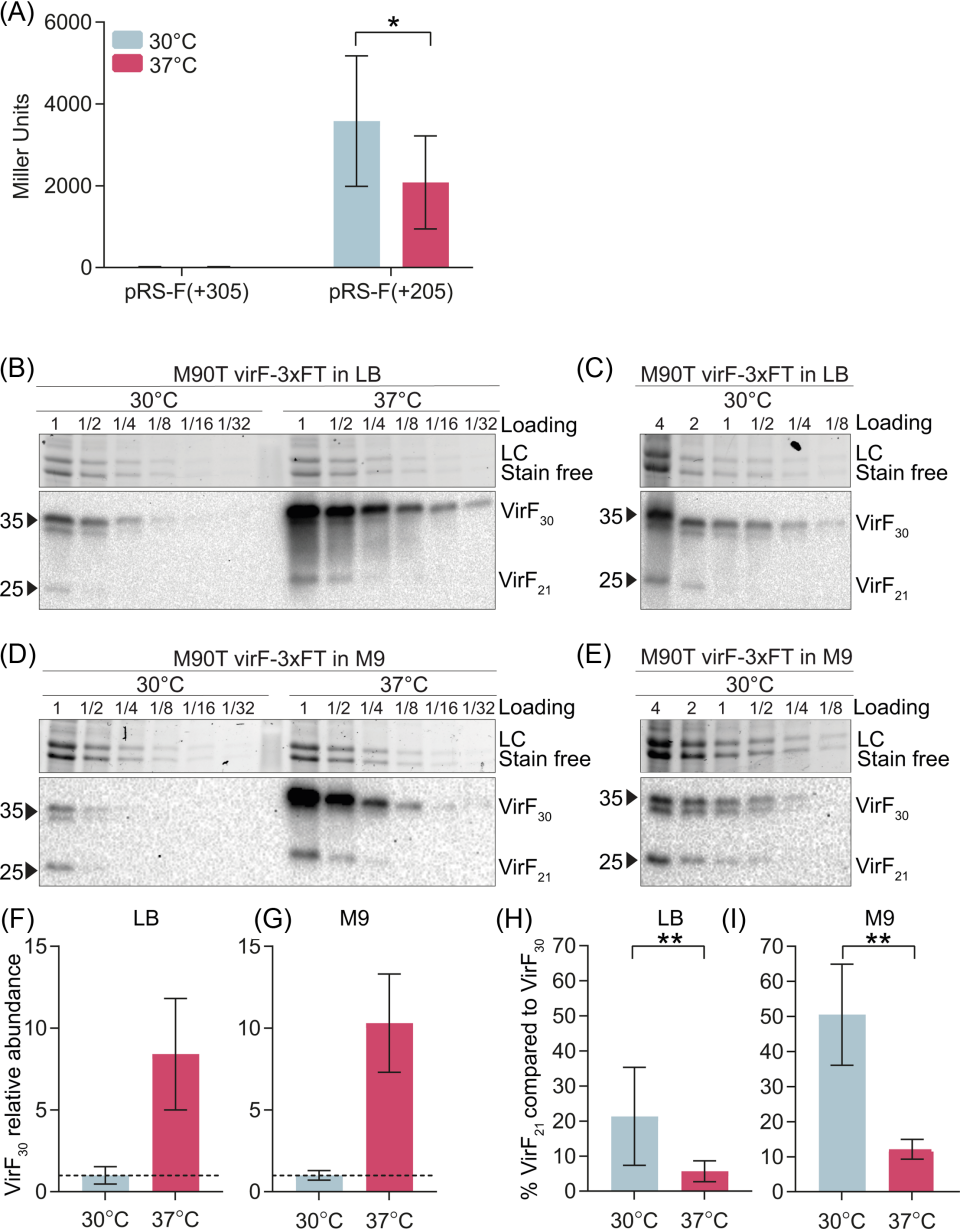
Low temperature (30°C) stimulates *virF_21_* llmRNA expression and an increased VirF_21_:VirF_30_ protein ratio. **(A)** ß-Galactosidase activity of the *virF-lacZ* transcriptional fusion pRS-F(+205) containing the internal promoter for the leaderless mRNA. The analysis was performed in *E.coli* DH10b. pRS-F(+305) was used as a negative control. The ß-Galactosidase activity was determined after subculture at 30 or 37°C. The activity is reported in Miller Units and represents the mean and standard deviation of 7 (pRS-F(+305)) and 13 (pRS-F(+205)) biological replicates from 3 different experiments. Statistical significance, comparing the ß-Galactosidase activity of the pRS-F(+205) fusion at 30 or 37°C, was determined by Mann-Whitney U test, *P < 0.05. **(B)** Detection of VirF_30_ and VirF_21_ at 30° and 37°C in protein extracts of the *Shigella* M90T strain carrying *virF-3xFT* grown in LB medium. A representative western blot with serial dilutions of the protein extracts is shown. **(C)** Detection of VirF_30_ and VirF_21_ at 30°C in protein extracts of the *Shigella* M90T strain carrying *virF-3xFT* grown in LB medium. A representative western blot in which protein extracts were concentrated to facilitate quantification is shown. **(D)** Detection of VirF_30_ and VirF_21_ at 30° and 37°C in protein extracts of the *Shigella* M90T strain carrying *virF-3xFT* grown in M9 medium. A representative western blot with serial dilutions of the protein extracts is shown. **(E)** Detection of VirF_30_ and VirF_21_ at 30°C in protein extracts of the *Shigella* M90T strain carrying *virF-3xFT* grown in M9 medium. A representative western blot in which protein extracts were concentrated to facilitate quantification is shown. **(F)** The relative VirF_30_ content in the *Shigella* M90T strain carrying *virF-3xFT* grown in LB at 30° and 37°C was determined by quantification of western blots of serially diluted samples. VirF_30_ level at 30°C was set as 1. Shown is the mean and standard deviation of 3 independent experiments. **(G)** The relative VirF_30_ content in the *Shigella* M90T strain carrying *virF-3xFT* grown in M9 medium at 30° and 37°C was determined by quantification of western blots of serially diluted samples. VirF_30_ level at 30°C was set as 1. Shown is the mean and standard deviation of 4 independent experiments. **(H)** VirF_21_ levels in the *Shigella* M90T strain carrying *virF-3xFT* grown in LB at 30°C and 37°C were quantified from western blots of concentrated samples. VirF_21_ content was quantified in comparison with the VirF_30_ content. Shown is the mean and standard deviation of 8 (30°C) and 6 (37°C) independent experiments. Statistical significance was determined by a Mann Whitney U test, **P < 0.01. **(I)** VirF_21_ levels in the *Shigella* M90T strain carrying *virF-3xFT* grown in M9 medium at 30°C and 37°C were quantified from western blots of concentrated samples. VirF_21_ content was quantified in comparison with the VirF_30_ content. Shown is the mean and standard deviation of 5 independent experiments. Statistical significance was determined by Mann–Whitney U test, **P < 0.01.

Interestingly, we found that the ß-galactosidase activity of the pRS-F (+205) fusion was significantly higher at 30°C than at the permissive host temperature of 37°C (Fig. 1A). Translation of both VirF forms in *Shigella flexneri* M90T was observed at both temperatures (Fig. 1B-E). In agreement with the positive regulation of *virF* expression at 37°C, VirF_30_ protein abundance was ∼8–10-fold higher at 37°C than at 30°C both in LB (Fig. 1B-C-F) and in M9 medium (Fig. 1D-E-G). Notably, the VirF_21_:VirF_30_ protein ratio differed dramatically between the two temperatures. While VirF_21_ represented ∼5–10% of the VirF_30_ protein content at 37°C (∼5% in LB; ∼ 10% in M9), it reached peaks of ∼20–50% at 30°C (∼20% in LB; ∼ 50% in M9) (Fig. 1C-E-H-I).

Taken together these results suggest that while *virF_21_* llmRNA transcription may be affected by several different stimuli, a high VirF_21_:VirF_30_ protein ratio is favoured at environmental temperature, rather than under host-like conditions. In this context, VirF_21_ might function as a molecular brake to minimize fitness costs when the *Shigella* virulence program is not required or undesired.

The *virF* genetic arrangement leading to the transcription and translation of two proteins from a single gene is not an isolated example. The *E.coli* copper chaperone CopA and the *Salmonella* LysR-type regulator LtrR also display transcription of two mRNA molecules and the translation of two protein forms under specific environmental conditions (Drees *et al*. 2017, Rebollar-Flores *et al*. 2020). Furthermore, computational analysis aimed at discovering overlooked regulatory elements showed that gene internal promoters are often associated with horizontally transferred genes, both in *E.coli* and in some archaeal species (Ten-Caten, Vêncio *et al*. 2018). This is particularly relevant here, since *virF* was horizontally acquired on the pINV during *Shigella´s* evolution towards pathogenicity (Yang *et al*. 2005). Altogether, this suggests the existence of a widespread adaptation strategy in bacteria to expand and diversify the protein repertoire and thereby optimize the response to changing external conditions.

### Ectopic expression of VirF_21_ at the permissive temperature reversibly suppresses the *Shigella* host cell invasive program

VirF_30_ activity governs the transition of *Shigella* between non-invasive and invasive states. When switching from 30°C to 37°C, already a modest increase in *virF* transcription is sufficient for full activation of the downstream virulence cascade and a host cell invasive phenotype (Le Gall *et al*. 2005). To test the hypothesis that ViF_21_ expression can prevent switching to the invasive phenotype, we explored the consequences of elevating VirF_21_ protein levels under the permissive temperature (37°C).

**Figure 2.**
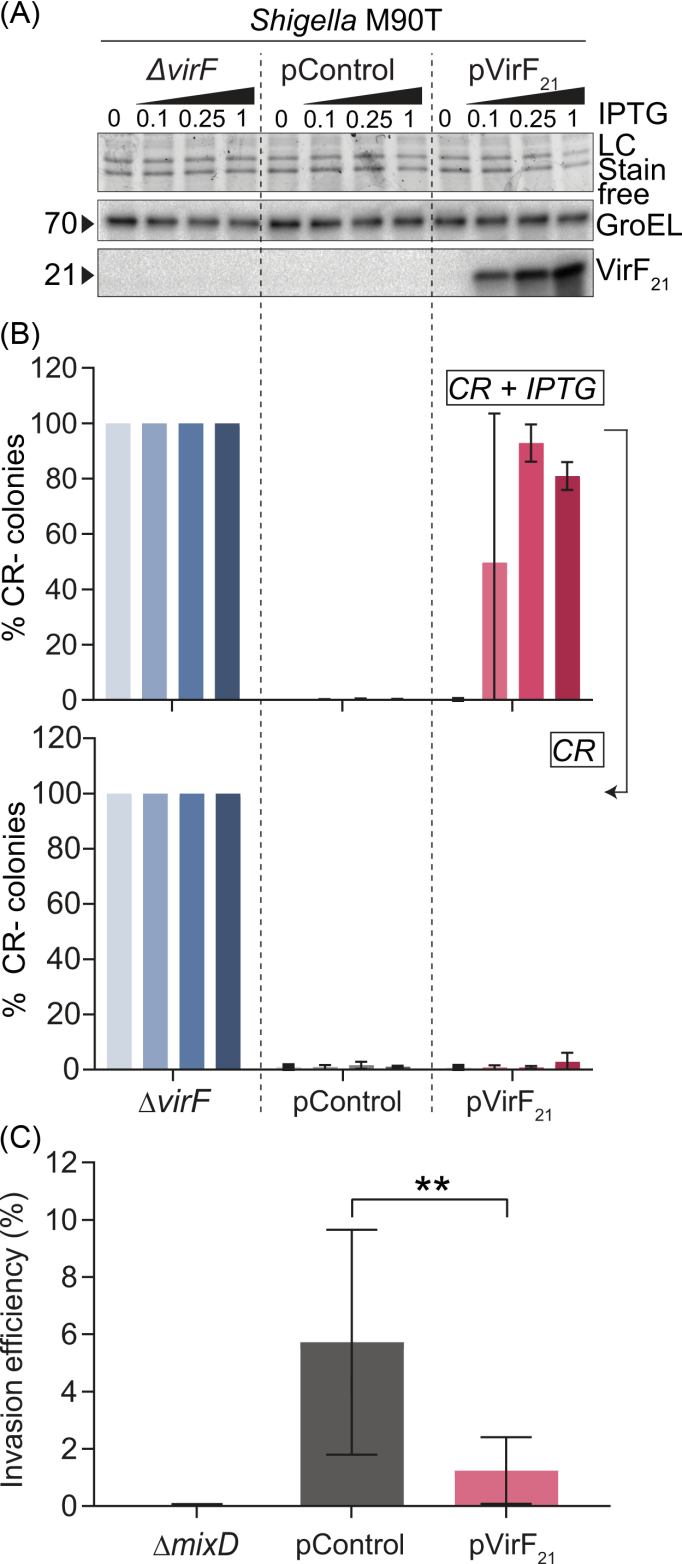
Elevated expression of VirF_21_ suppresses *Shigella* virulence at 37°C. **(A)** Western blot with VirF antibodies on extracts from a *Shigella* M90T *ΔvirF* mutant and M90T strains harbouring pControl (empty vector) or pVirF_21_, a plasmid carrying the *virF_21_* coding sequence under an inducible pTaq promoter. The strains were grown in the presence of increasing concentration of IPTG (0, 0.1, 0.25, 1 mM) to induce VirF_21_ expression. GroEL protein was detected and used as internal loading control. A loading control using the Stain free method is also shown. **(B)** (upper panel) % CR- colonies upon spreading of the indicated strains on CR plates containing increasing concentrations of IPTG (0, 0.1, 0.25, 1 mM) to induce VirF_21_ expression. (bottom panel) % CR- colonies upon scraping and re-plating of the colonies obtained on the previous plates, onto new CR plates without IPTG selection. Data come from at least three replicates from two independent experiments. ∼200–600 colonies/replicate were examined for the CR phenotype. **(C)** Invasion efficiency of the indicated *Shigella* M90T strains in sub-confluent Caco-2 cell layers. Cells were infected at MOI 100 for 1h, and analysed by selective plating of intracellular bacteria. Shown are CFU data expressed as the percentage of the inoculum retrieved in the intracellular population. Shown are CFU data for 7 (M90T pControl) and 9 (M90T *ΔmxiD*, M90T pVirF_21_) biological replicates from 3 independent experiments. Bars represent mean and standard deviation. Statistical significance was determined by Mann Whitney U test, **P < 0.01.

First, we investigated the effect of ectopic VirF_21_ expression on the ability of *Shigella* to bind Congo Red (CR), a phenotype linked to virulence and the presence of *virF*, resulting in red colonies (CR+) on solid medium (Sakai *et al*. 1986a). *Shigella flexneri* M90T was transformed with either a plasmid that allowed IPTG-inducible expression of VirF_21_ (pVirF_21_; carries the Ptac promoter; reported as pAC-21 in Di Martino *et al*. 2016b), or the corresponding empty vector (pControl; previously named pGIP7 in Falconi *et al*. 2001). Ectopic expression of VirF_21_ was detected in the presence of graded concentrations of IPTG (0.1–0.25–1 mM), but not in the absence of IPTG (Fig. 2A). We plated dilutions of exponential cultures of M90T pControl, M90T pVirF_21_ and M90T *ΔvirF* on CR plates containing increasing concentrations of IPTG and incubated at 37°C. Fig. 2B and figure S2 show that IPTG-induced ectopic expression of VirF_21_ led to the appearance of a high percentage (∼50–90%) of white (CR-) colonies, reaching comparable levels as the non-virulent M90T *ΔvirF* mutant, a strain known to exhibit a completely CR- phenotype (Sakai *et al*. 1986b). In particular, a robust CR- phenotype (>80% white colonies) was observed on CR plates containing 0.25 and 1 mM IPTG, while the percentage white colonies was somewhat variable in the presence of 0.1 mM IPTG. This observation suggests a borderline VirF_21_ expression at IPTG concentrations lower than 0.25 mM. Importantly, when the CR- M90T pVirF_21_ colonies were collected from the plates supplemented with IPTG and re-plated on new CR plates devoid of the IPTG inducer, the bacteria reverted back to virtually exclusively CR + colonies (Fig. 2B; <3% CR-). These results suggest that CR binding is subjected to a VirF_21_-driven reversible switch, linked also to a decrease in virulence gene expression (Fig S3A-B; and Di Martino *et al*. 2016b).

Next, we infected human colonic epithelial Caco-2 cells with the M90T pControl and M90T pVirF_21_ strains, to test how ectopic VirF_21_ expression impacts *Shigella* host cell invasion. M90T *ΔmxiD* (lacking the outer membrane ring MxiD protein, resulting in a nonfunctional T3SS) was used as a non-invasive control strain. To ensure robust VirF_21_ expression with minimal side effects, we induced VirF_21_ expression with the intermediate concentration of IPTG (0.25 mM, as informed by the CR binding assay; Fig. 2A and B). As expected, the M90T *ΔmxiD* strain failed at infecting Caco-2 cells (Fig. 2C). Notably, ectopic expression of VirF_21_ (M90T pVirF_21_) led to a ∼4–5-fold decrease in *Shigella*´s ability to infect Caco-2 cells, as compared to the M90T pControl strain (Fig. 2C).

Taken together, these results show that elevated VirF_21_ expression at 37°C can suppress the *Shigella* invasive program, signified by a CR-phenotype on plates, reduced virulence gene expression, and hampered capacity to infect epithelial cells. Considering that VirF_21_ makes up a larger fraction of the total VirF protein pool at 30°C than at 37°C (Fig. 1), the above results reinforce the hypothesis that VirF_21_ may negatively tune *Shigella* virulence gene expression, when this is undesirable. It is known that the pINV virulence plasmid is subjected to high counter-selective pressure at 37°C. With increasing number of generations, mutations, insertions of IS sequences and/or complete loss of the virulence cascade top regulators *virF* or *virB* occur at 37°C (Sasakawa *et al*. 1986, Schuch and Maurelli 1997, Pilla *et al*. 2017). Occasionally, the selective pressure can escalate leading even to the loss, or integrational silencing, of the entire pINV (Zagaglia *et al*. 1991, Pilla *et al*. 2017). At 30°C, virulence gene expression is silenced and therefore the selective pressure on the pINV is relieved, resulting in minimal loss or mutations (Schuch and Maurelli 1997). In this context, it is tempting to speculate that VirF_21_ expression represents an additional regulatory layer to minimize virulence gene expression leakage and therefore promote overall pINV stability under certain environmental conditions.

### The C-terminal region of VirF_21_ is required for negative regulation of *Shigella* host cell invasion

The experimental setting based on ectopic VirF_21_ expression precludes assessment of the impact of endogenous VirF_30_ and VirF_21_ levels expressed from their native genetic context. Despite significant efforts, we have however been unsuccessful at generating a *Shigella flexneri* scarless mutant expressing VirF_30_ protein only from the endogenous locus. This may suggest that the native *virF* locus sequence is unusually intolerant to perturbation, although we cannot formally rule out other technical explanations.

To better understand the relationship between VirF_21_ sequence and function, and to verify the specificity of the above results, we therefore opted for a site directed mutagenesis approach, targeting the untagged *virF_21_* gene cloned into the pVirF_21_ plasmid. VirF_30_ and VirF_21_ belong to the family of AraC-like transcriptional regulators (Cortés-Avalos *et al*. 2021). This group comprises both positive and negative transcriptional regulators, which often control virulence systems across different gram-negative bacterial species (i.e. *Salmonella*, *Yersinia*, *Vibrio cholera*, (Gallegos *et al*. 1997, Cortés-Avalos *et al*. 2021)). The mechanisms governing AraC-like protein expression and regulation have been successfully studied in many cases. However, their biochemical and structural properties have been less well characterized, since AraC-like proteins are often difficult to purify (Cortés-Avalos *et al*. 2021). The DNA sequences targeted by VirF_30_ have nevertheless been identified in some cases (i.e. within *icsA*, RNAG, and *virB* promoters) (Tobe *et al*. 1993, Giangrossi *et al*. 2010, Tran *et al*. 2011), and the VirF_21_ binding site within the *virF* promoter was also previously mapped (Di Martino *et al*. 2016b). VirF_21_ and VirF_30_ share the C-terminal portion, which contains the two typical AraC-like Helix-Turn-Helix (HTH) DNA binding motifs, separated by an alfa helix linker. VirF_21_ lacks the N-terminal domain of VirF_30_, which is believed to have oligomerization properties. Some of the amino acids likely involved in the interaction between VirF_30_ and its DNA targets have been identified by a combined random and site directed mutagenesis approach (Porter and Dorman 2002).

**Figure 3.**
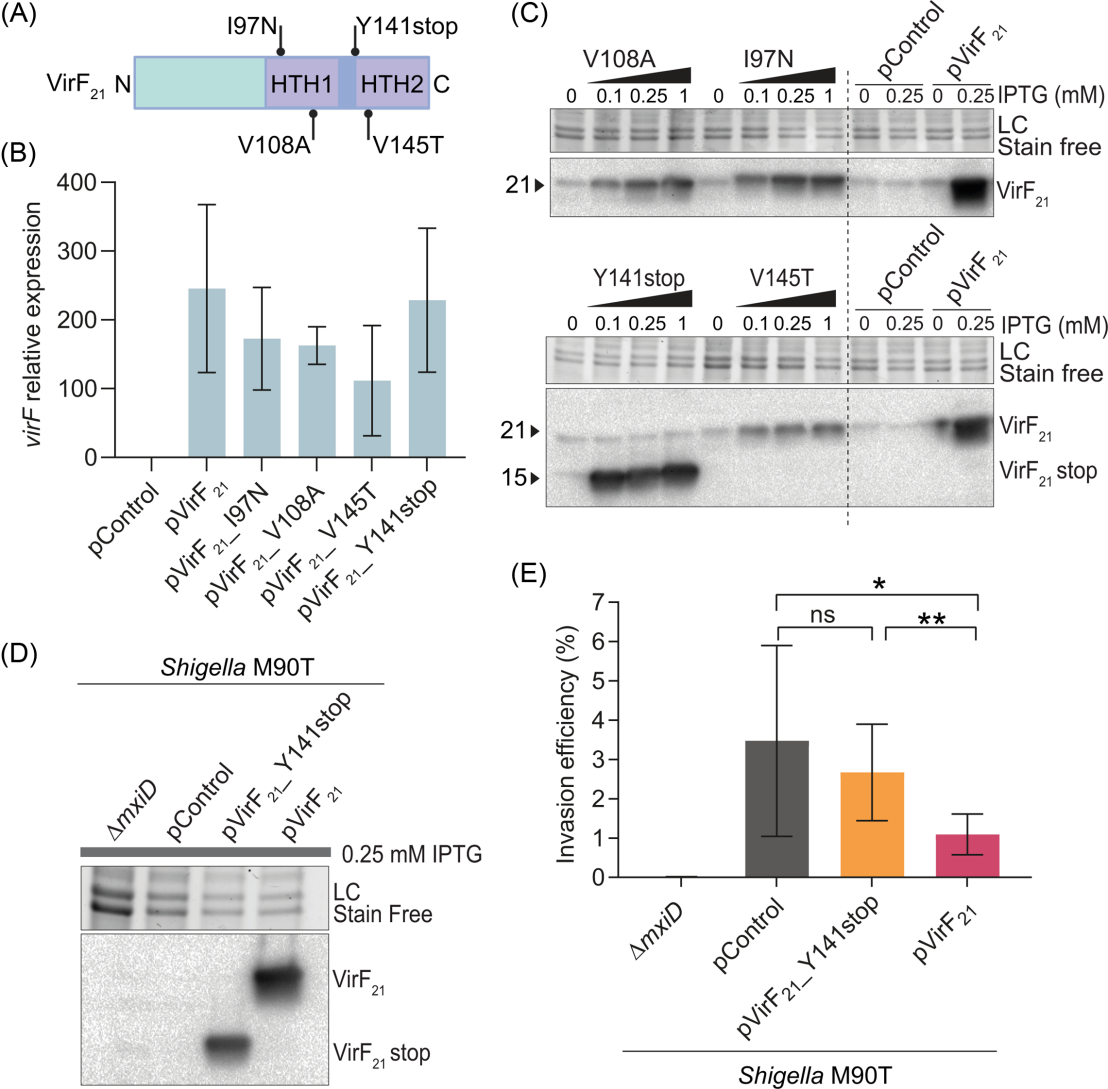
The C-terminal HTH2 motif is required for VirF_21_ function. **(A)** Schematic representation of the VirF_21_ protein sequence, with relevant mutagenized amino acid positions indicated. **(B)***virF* mRNA expression levels (2^−ΔΔCt^) as a function of protein induction with 0.25 mM IPTG in *Shigella* M90T strains harbouring pControl, pVirF_21_, pVirF_21__I97N, pVirF_21__V108A, pVirF_21__V145T, or pVirF_21__ Y141stop. Data come from 6–7 biological replicates from 3 independent experiment and were normalized to the *virF* expression in the M90T pControl strain. **(C)** VirF_21_ protein levels detected by western blot as a function of IPTG induction (increasing concentration: 0, 0.1, 0.25, 1 mM) in *Shigella* M90T strains harbouring pControl, pVirF_21_, pVirF_21__I97N, pVirF_21__V108A, pVirF_21__V145T, or pVirF_21__141stop. VirF_21__Y141stop produces a smaller protein (∼ 15kDa), since the last 39 aa in the C-terminal part are deleted. **(D)** VirF_21_ protein levels detected by western blot in a *Shigella* M90T *ΔmxiD* mutant and in *Shigella* M90T strains harbouring pControl, pVirF_21_, or pVirF_21__Y141stop plasmids in the presence of 0.25 mM IPTG. **(E)** Invasion efficiency of the indicated *Shigella* M90T strains in sub-confluent Caco-2 cell layers. Cells were infected at MOI 100 for 1h, and analysed by selective plating of intracellular bacteria. Shown are CFU data expressed as the percentage of the inoculum retrieved in the intracellular population. Shown are CFU data for 4 (*ΔmxiD)* and 6 (*Shigella* M90T strains harbouring pControl, pVirF_21_, or pVirF_21__Y141stop plasmids) biological replicates from 2 independent experiments. Bars represent mean and standard deviation. Statistical significance was determined by Mann Whitney U test, ns = non significant, *P < 0.05, **P < 0.01.

In an attempt to obtain a non-functional VirF_21_, we transplanted an assortment of mutations shown to affect VirF_30_ function in the prior study (Porter and Dorman 2002). The following mutations were introduced onto the pVirF_21_ plasmid: I97N, V108A, V145T and Y141stop, here reported considering VirF_30_-Met84 (Porter and Dorman 2002) = VirF_21_-Met1 (Fig. 3A; Di Martino et al 2016b; previously reported in Porter and Dorman 2002 as I180N, V191A, V228T, and Y224Och respectively). The substitutions I97N and V108A target the HTH1 DNA binding motif, while the V145T substitution is located within the HTH2 DNA binding motif. Finally, the deletion of the HTH2 motif was achieved by introducing a stop codon at position 141 (Y141stop; deletion of 39 aa in the C-terminus). Upon induction with IPTG in *Shigella flexneri* M90T, the wild type (wt) and the mutated versions of *virF_21_* showed broadly similar transcriptional levels (Fig. 3B). However, VirF_21_ protein levels (monitored by a halon anti-VirF antibody) were markedly lower for the VirF_21__I97N, VirF_21__V108A and VirF_21__V145T mutant constructs than in the VirF_21__wt carrying strain (Fig. 3C), suggesting a possible impact of these amino acid substitutions on protein stability. Only the truncated VirF_21__Y141stop construct generated protein levels comparable to the strain harbouring the pVirF_21__wt plasmid (Fig. 3C-D). Next, we infected Caco-2 cells with the strains ectopically expressing either VirF_21__wt (pVirF_21_) or VirF_21__stop (pVirF_21__ Y141stop). As evident from Fig. 3E, the *Shigella* pVirF_21__Y141stop strain retained the ability to infect Caco-2 cells at similar levels as the control strain (*Shigella* pControl), while the *Shigella* pVirF_21__wt strain again showed a ∼3 fold lower invasion capacity (Fig. 3E; compare also with Fig. 2C). These results show that the VirF_21__stop protein, lacking the predicted DNA binding HTH2 motif, can be expressed to similar levels as full-length VirF_21_, but is non-functional. This validates the specificity of the VirF_21_ suppressive effects observed in the above experiments (Fig. 2), and reveals a key role of the C-terminal portion for VirF_21_ functionality.

## Conclusions

Pathogenic bacteria are masters at adapting to fast-changing environmental cues. *Shigella* encounters many different environmental conditions and switches flexibly between extracellular and intracellular lifestyles. While the activation of the *virF* regulatory cascade is a crucial event for *Shigella* expression of the invasive program (Schroeder and Hilbi 2008), it also constitutes a significant fitness cost for the bacterial population (Schuch and Maurelli 1997). Thus, it is not surprising that *Shigella* employs a multi-layered regulatory arsenal to ensure expression of the virulence genes only when these are needed. VirF_21_ has been identified as a possible negative autoregulator of VirF_30_, but the impact on the invasive *Shigella* phenotype had not been addressed (Di Martino *et al*. 2016b). Our results expand on these previous findings, by illustrating that *virF_21_* llmRNA expression and the VirF_21_:VirF_30_ protein ratio is enhanced at 30°C, a common condition *Shigella* encounters outside of the host. In this context, it seems plausible that VirF_21_ serves to suppress virulence gene expression when not desired. Indeed, when ectopically expressed, VirF_21_ is capable of suppressing the *Shigella* virulence program at the permissive temperature 37°C, resulting in a CR- phenotype on plates, lowered levels of virulence gene transcripts, and an impaired ability to infect host cells. This suppressive activity requires the HTH2-motif-containing C-terminus of the VirF_21_ protein.

While the physiological impact of VirF_21_ remains to be completely explored under the multitude of possible environmental conditions, the findings presented here highlight the interconnected mechanisms that ensure fine-tuned regulation of virulence properties across the *Shigella* life cycle.

## Supplementary Material

fnac043_Supplemental_File
