# Supplementary material for: The VirF21:VirF30 protein ratio is affected by temperature and impacts Shigella flexneri host cell invasion
Source: FEMS Microbiol Lett. 2022 May 6;369(1):fnac043. doi: 10.1093/femsle/fnac043 (PMC9217107; doi:10.1093/femsle/fnac043)
Supplement: fnac043_Supplemental_File [file fnac043_supplemental_file.pdf]

Supplementary material

The $VirF_{21}$:$VirF_{30}$ protein ratio is affected by temperature and impacts *Shigella flexneri* host cell invasion

Eva Skovajsová[1], Bianca Colonna[2], Gianni Prosseda[2], Mikael E. Sellin[1], Maria Letizia Di Martino[1,]*

[1]Science for Life Laboratory, Department of Medical Biochemistry and Microbiology, Uppsala University, Sweden

[2]Department of Biology and Biotechnology "C. Darwin", Istituto Pasteur Italia, Sapienza Università di Roma, Rome, Italy

*Correspondence: M.L.D.M ml.dimartino@imbim.uu.se

Full address of corresponding author: Maria Letizia Di Martino, Uppsala University, Department of Medical Biochemistry and Microbiology, Box 582, 75 123 Uppsala, Sweden, Phone: +46722092710

Figure S1

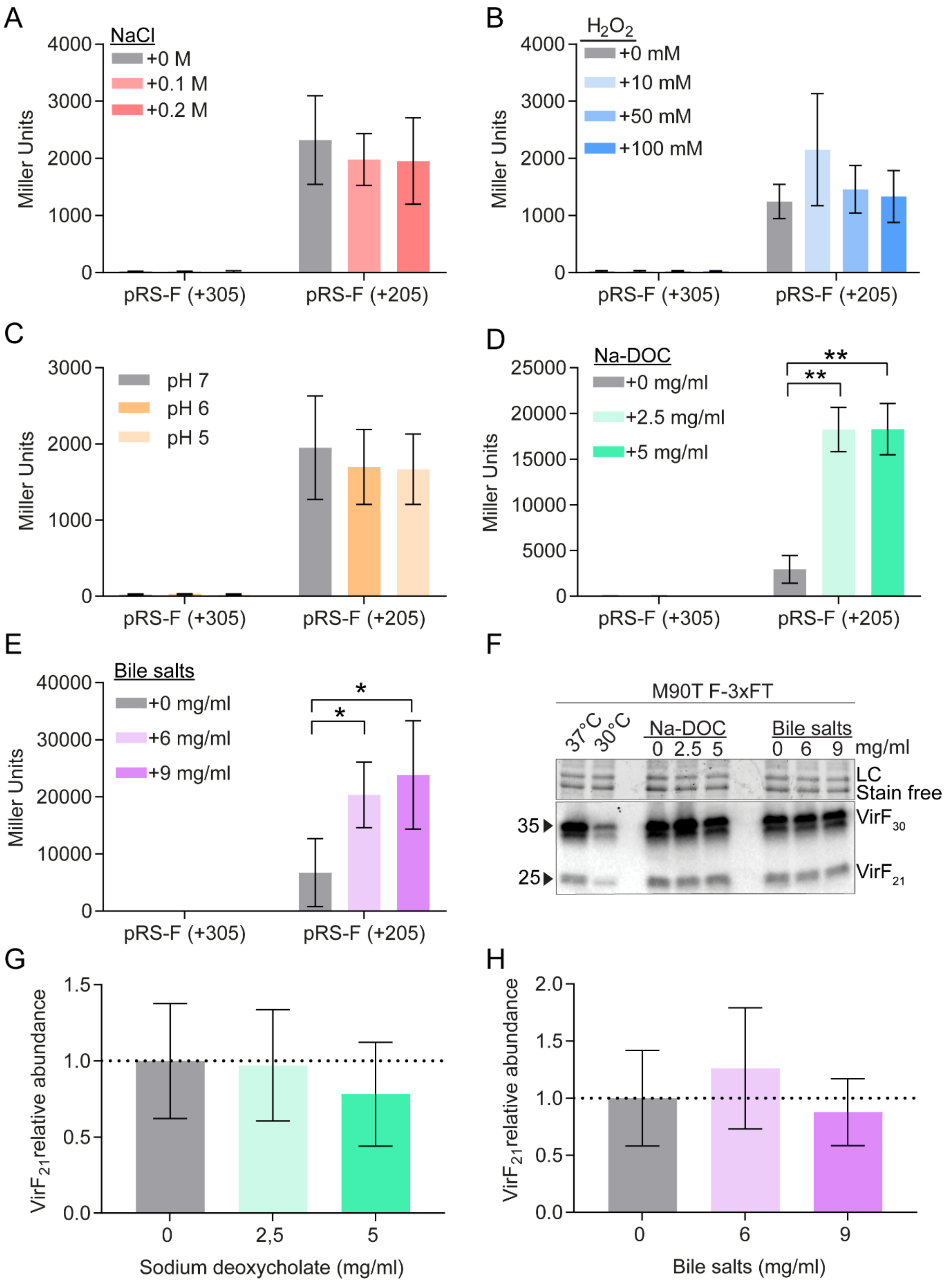

Figure S1. Intestinal environment-like conditions can alter *virF₂₁* llmRNA expression, but have no detectable effect on VirF₂₁ protein levels. A. ß-galactosidase activity of *virF-lacZ* transcriptional fusion pRS-F(+205) containing the internal promoter for the leaderless mRNA. The analysis was performed in *E.coli* DH10b. pRS-F(+305) was used as a negative control. The ß-galactosidase activity

was determined after subculture in M9 medium supplemented with 0, 0.1 or 0.2M NaCl. The activity is reported in Miller Units and represents the mean and standard deviation of 5 biological replicates from 2 different experiments. **B.** ß-galactosidase activity of *virF-lacZ* transcriptional fusions pRS-F(+205) and pRS-F(+305) as in A. The ß-galactosidase activity was determined after subculture in M9 medium supplemented with 0, 10, 50 or 100mM hydrogen peroxide (H_2O_2). Data represent the mean and standard deviation of 5 biological replicates from 2 different experiments. **C.** ß-galactosidase activity of *virF-lacZ* transcriptional fusions pRS-F(+205) and pRS-F(+305) as in A. The ß-galactosidase activity was determined after subculture in M9 medium at pH 5, 6 or 7. Data represent the mean and standard deviation of 5 biological replicates from 2 different experiments. **D.** ß-galactosidase activity of *virF-lacZ* transcriptional fusions pRS-F(+205) and pRS-F(+305) as in A. The ß-galactosidase activity was determined after subculture in M9 medium supplemented with 0, 2.5 or 5mg/ml Sodium deoxycholate (Na-DOC). Data represent the mean and standard deviation of 5 biological replicates from 2 different experiments. Statistical significance was determined by Mann Whiney U test, ** $P < 0.01$. **E.** ß-galactosidase activity of *virF-lacZ* transcriptional fusions pRS-F(+205) and pRS-F(+305) as in A. The ß-galactosidase activity was determined after subculture in M9 medium supplemented with 0, 6 or 9mg/ml Bile Salts. Data represent the mean and standard deviation of 5 biological replicates from two independent experiments. Statistical significance was determined by Mann Whiney U test,* $P < 0.05$. **F.** Detection of $VirF_{30}$ and $VirF_{21}$ in protein extracts from the *Shigella* M90T strain carrying *virF-3XFT* grown in M9 medium supplemented with increasing concentration of Na-DOC (0, 2.5, 5mg/ml) or Bile salts (0, 6, 9mg/ml). Protein extracts from bacteria grown at 30°C or 37°C are also included for comparison. Loading control using the Stain free method is shown. **G.** The relative $VirF_{21}$ protein content was determined by quantification of western blots of serially diluted samples as in F. $VirF_{21}$ level in the untreated samples was set as 1. Shown is the mean and standard deviation of 5 independent experiments. **H.** The relative $VirF_{21}$ protein content was determined by quantification of western blots of serially diluted samples as in F. $VirF_{21}$ level in the untreated samples was set as 1. Shown is the mean and standard deviation of 5 independent experiments.

Figure S2

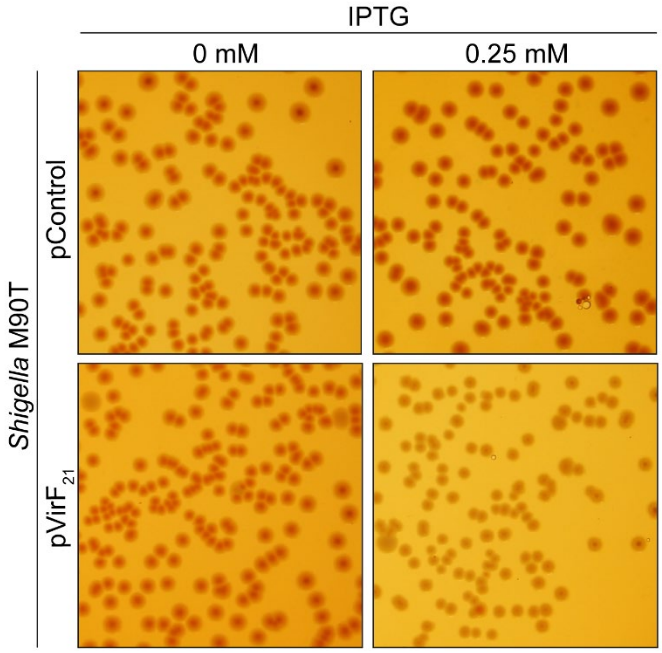

Figure S2. VirF$_{21}$ expression leads to a CR- *Shigella* phenotype. CR binding ability of *Shigella* M90T harbouring pControl (empty vector) or pVirF$_{21}$ (allows IPTG-inducible expression of VirF$_{21}$) upon spreading on CR plates with 0 or 0.25 mM IPTG. Representative images are shown.

Figure S3

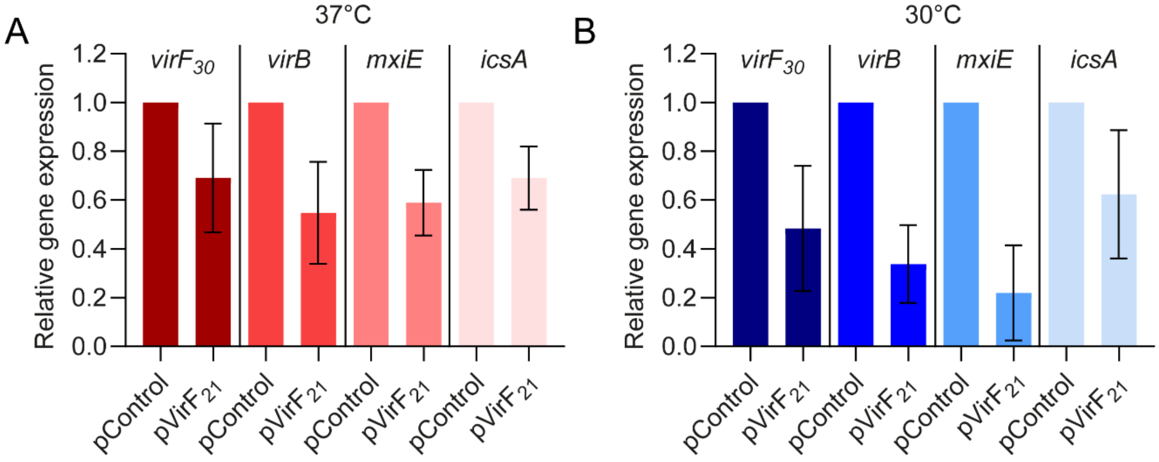

Figure S3. VirF$_{21}$ negatively regulates virulence gene expression. A. Expression levels of *virF$_{30}$, virB, mxiE* and *icsA* analysed in *Shigella* M90T strains upon induction of VirF$_{21}$ (pVirF$_{21}$) vs the control (pControl) at 37°C. Data were generated from 3 independent experiments and are presented as mean ± SD. A minimum of 2 technical replicates were run in each experiment. **B.** Expression levels of *virF$_{30}$, virB, mxiE* and *icsA* analysed in *Shigella* M90T strains upon induction of VirF$_{21}$ (pVirF$_{21}$) vs the control (pControl) at 30°C. Data were generated from 3 independent experiments and are presented as mean ± SD. A minimum of 2 technical replicates were run in each experiment.

Table S1. Strains and plasmids used in this study

Strain	Genotype	Reference
M90T	*S.flexneri* 5a, wt	(1)
M90T $^{\Delta mxiD}$	*S.flexneri* 5a, ΔmxiD	This study
M90T$^{\Delta virF}$	*S.flexneri* 5a, ΔvirF	(2)
M90T-F-3xFT	*S.flexneri* 5a, virF-3xFT	(2)
DH10b	*E. coli* K12	(3)
**Plasmids**	**Description**	**Reference**
pRS-F(+305)	*virF-lacZ* transcriptional fusion in pRS415 (pos: +305 to +405)	(4)
pRS-F(+205)	*virF-lacZ* transcriptional fusion in pRS415 (pos: +205 to +405)	(4)
pVirF$_{21}$	pGIP7 derivative carrying virF21 ORF	(4)
pControl	Previously named pGIP7; pACYC184 derivative carrying lacI gene and Plac	(5)
pVirF$_{21}$_I97N	pVirF$_{21}$ derivative with ATT to AAT mutation at position 289-291 of virF$_{21}$ coding sequence	This study
pVirF$_{21}$_V108A	pVirF$_{21}$ derivative with GTT to GCC mutation at position 322-324 of virF$_{21}$ coding sequence	This study
pVirF$_{21}$_V145T	pVirF$_{21}$ derivative with GTA to ACC mutation at position 433-435 of virF$_{21}$ coding sequence	This study
pVirF$_{21}$_Y141Stop	pVirF$_{21}$ derivative with TAT to TAA mutation at position 421-423 of virF$_{21}$ coding sequence	This study

REFERENCES

1. Sansonetti PJ, d'Hauteville H, Formal SB, Toucas M. 1982. Plasmid-mediated invasiveness of "Shigella-like" Escherichia coli. Ann Microbiol (Paris) 133:351–5.

2. Leuzzi A, Di Martino ML, Campilongo R, Falconi M, Barbagallo M, Marcocci L, Pietrangeli P, Casalino M, Grossi M, Micheli G, Colonna B, Prosseda G. 2015. Multifactor Regulation of the MdtJI Polyamine Transporter in Shigella. PLoS One 10:e0136744.

3. Durfee T, Nelson R, Baldwin S, Plunkett G, Burland V, Mau B, Petrosino JF, Qin X, Muzny DM, Ayele M, Gibbs RA, Csörgo B, Pósfai G, Weinstock GM, Blattner FR. 2008. The complete genome sequence of Escherichia coli DH10B: insights into the biology of a laboratory workhorse. J Bacteriol 190:2597–606.

4. Di Martino ML, Romilly C, Wagner EG, Colonna B, Prosseda G. 2016. One Gene and Two Proteins: a Leaderless mRNA Supports the Translation of a Shorter Form of the Shigella VirF Regulator. mBio. 7(6):e01860-16.

5. Falconi M, Prosseda G, Giangrossi M, Beghetto E, Colonna B. 2001. Involvement of FIS in the H-NS-mediated regulation of virF gene of Shigella and enteroinvasive Escherichia coli. Mol Microbiol 42:439–52.

Table S2. Primers and PCR products used in this study

Primer	Sequence (5'-3')
mxiD_delF	GACTCTCTTGATTGTATTGTTACCATTGATAGTTAATGCTGTGTAGG<br>CTGGAGCTGCTTC
mxiD_delR	CATACATCACCTACTTTATAGTTAGTAATTTAAGTATGAACATATGA<br>ATATCCTCCTTA
virFQF	GCCTTTCGGCAAAAGAAAGAT
virFQR	TCGAAGTATATAAAGCTTCCTCATCAGA
virF30QF	CAATTTGCCCTTCATCGATAGTC
virF30QR	AAAGGTGTTCAATGACGGTTAGC
virBQF	CCAAGTTCTCGGATGCTATGC
virBQR	CTCTTGATGCCAGAAAACTAGCAA
icsAQF	TGATGGACTTTCTCCCTTGGG
icsAQR	TACCACGCATCCATTCCATCT
mxiEQF	AACACTGAGTGGGTGTGCTTT
mxiEQR	CCATGAAGCACCCATCTACTT
nusAQF	TGAAGCCGCACGTTATGAAG
nusAQR	TCAACGTAATTCGCCCAGGTT
PCR product	Sequence
virF21_I97N	gttttcacgagcacttcaccaacaaggaccatagcccggggatccATGGAACCAATTTATTCA<br>TTTCAACACTCCTATTCTGAGGAGAAAAGGGGGTTAAACAAAAAAA<br>TATTCCTCCTCTCTGAGGAGGAGGTTTCTATCGATTTGTTCAAATCT<br>ATAAAAGAGATGCCTTTCGGCAAAAGAAAGATCTATAGTTTAGCTT<br>GCCTTTTATCAGCTGTTTCTGATGAGGAAGCTTTATATACTTCGATA<br>TCGATAGCTTCTTCTCTTAGTTTTTCTGATCAGATAAGGAAGATTGT<br>TGAAAAAAACATCGAGAAGAGATGGCGTCTTTCTGATAATTCAAAT<br>AACTTGAATTTATCAGAAATAGCTGTTAGAAAACGATTGGAGAGTG<br>AAAAATTAACATTTCAACAAATCCTTCTTGATATTCGCATGCATCAT<br>GCAGCAAAGCTTTTATTGAATAGTCAAAGCTATATTAATGATGTATC<br>AAGACTTATCGGAATATCAAGCCCATCTTATTTTATAAGGAAATTTA<br>ATGAATATTATGGTATAACTCCAAAGAAATTTTACTTATATCATAAA<br>AAATTTTAAATGCTTCATAGCCCATCGCTATTGCCAGATGGGTTTTC<br>Cggatccacaggacgggtgtggtcgccatgatcgcgtagtcgatag
virF21_V108A	gttttcacgagcacttcaccaacaaggaccatagcccggggaTCCATGGAACCAATTTATTC<br>ATTTCAACACTCCTATTCTGAGGAGAAAAGGGGGTTAAACAAAAAA<br>ATATTCCTCCTCTCTGAGGAGGAGGTTTCTATCGATTTGTTCAAATC<br>TATAAAAGAGATGCCTTTCGGCAAAAGAAAGATCTATAGTTTAGCT<br>TGCCTTTTATCAGCTGTTTCTGATGAGGAAGCTTTATATACTTCGAT<br>ATCGATAGCTTCTTCTCTTAGTTTTTCTGATCAGATAAGGAAGATTG<br>TTGAAAAAAACATCGAGAAGAGATGGCGTCTTTCTGATATTTCAAA<br>TAACTTGAATTTATCAGAAATAGCTGCCAGAAAACGATTGGAGAGT<br>GAAAAATTAACATTTCAACAAATCCTTCTT<br>GATATTCGCATGCATCATGCAGCAAAGCTTTTATTGAATAGTCAAA<br>GCTATATTAATGATGTATCAAGACTTATCGGAATATCAAGCCCATCT<br>TATTTTATAAGGAAATTTAATGAATATTATGGTATAACTCCAAAGA<br>AATTTTACTTATATCATAAAAAATTTTAAATGCTTCATAGCCCATCG<br>CTATTGCCAGATGGGTTTTCCggatccacaggacgggtgtggtcgccatgatcgcgtagtcg<br>atag
virF21_V145T	gttttcacgagcacttcaccaacaaggaccatagcccggggaTCCATGGAACCAATTTATTC<br>ATTTCAACACTCCTATTCTGAGGAGAAAAGGGGGTTAAACAAAAAA<br>ATATTCCTCCTCTCTGAGGAGGAGGTTTCTATCGATTTGTTCAAATC<br>TATAAAAGAGATGCCTTTCGGCAAAAGAAAGATCTATAGTTTAGCT

	TGCCTTTTATCAGCTGTTTCTGATGAGGAAGCTTTATATACTTCGATATCGATAGCTTCTTCTCTTAGTTTTTCTGATCAGATAAGGAAGATTGTTGAAAAAAACATCGAGAAGAGATGGCGTCTTTCTGATATTTCAAATAACTTGAATTTATCAG<br>AAATAGCTGTTAGAAAACGATTGGAGAGTGAAAAATTAACATTTCAACAAATCCTTCTTGATATTCGCATGCATCATGCAGCAAAGCTTTTATTGAATAGTCAAAGCTATATTAATGATACCTCAAGACTTATCGGAATATCAAGCCCATCTTATTTTATAAGGAAATTTAATGAATATTATGGTATAACTCCAAAGAAATTTTACTTATATCATAAAAAATTTTAAATGCTTCATAGCCCATCGCTATTGCCAGATGGGTTTTCCggatccacaggacgggtgtggtcgccatgatcgcgtagtcgatag
virF21_Y141Stop	gttttcacgagcacttcaccaacaaggaccatagcccggggaTCCATGGAACCAATTTATTCATTTCAACACTCCTATTCTGAGGAGAAAAGGGGGTTAAACAAAAAAATATTCCTCCTCTCTGAGGAGGAGGTTTCTATCGATTTGTTCAAATCTATAAAAGAGATGCCTTTCGGCAAAAGAAAGATCTATAGTTTAGCTTGCCTTTTATCAGCTGTTTCTGATGAGGAAGCTTTATATACTTCGATATCGATAGCTTCTTCTCTTAGTTTTTCTGATCAGATAAGGAAGATTGTTGAAAAAAACATCGAGAAGAGATGGCGTCTTTCTGATATTTCAAATAACTTGAATTTATCAGAAATAGCTGTTAGAAAACGATTGGAGAGTGAAAAATTAACATTTCAACAAATCCTTCTTGATATTCGCATGCATCATGCAGCAAAGCTTTTATTGAATAGTCAAAGCTAAATTAATGATGTATCAAGACTTATCGGAATATCAAGCCCATCTTATTTTATAAGGAAATTTAATGAATATTATGGTATAACTCCAAAGAAATTTTACTTATATCATAAAAAATTTTAAATGCTTCATAGCCCATCGCTATTGCCAGATGGGTTTTCCggatccacaggacgggtgtggtcgccatgatcgcgtagtcgatag
